# A Bittersweet Kiss of Gram-Negative Bacteria: The Role of ADP-Heptose in the Pathogenesis of Infection

**DOI:** 10.3390/microorganisms11051316

**Published:** 2023-05-17

**Authors:** Karolina Sidor, Tomasz Skirecki

**Affiliations:** Department of Translational Immunology and Experimental Intensive Care, Centre of Postgraduate Medical Education, Marymoncka 99/103, 01-813 Warsaw, Poland

**Keywords:** innate immunity, pathogen-associated molecular pattern PAMP, pattern recognition receptor PRR, lipopolysaccharide, TIFA, inflammation

## Abstract

Due to the global crisis caused by the dramatic rise of drug resistance among Gram-negative bacteria, there is an urgent need for a thorough understanding of the pathogenesis of infections of such an etiology. In light of the limited availability of new antibiotics, therapies aimed at host–pathogen interactions emerge as potential treatment modalities. Thus, understanding the mechanism of pathogen recognition by the host and immune evasion appear to be the key scientific issues. Until recently, lipopolysaccharide (LPS) was recognized as a major pathogen-associated molecular pattern (PAMP) of Gram-negative bacteria. However, recently, ADP-L-glycero-β-D-manno-heptose (ADP-heptose), an intermediate carbohydrate metabolite of the LPS biosynthesis pathway, was discovered to activate the hosts’ innate immunity. Therefore, ADP-heptose is regarded as a novel PAMP of Gram-negative bacteria that is recognized by the cytosolic alpha kinase-1 (ALPK1) protein. The conservative nature of this molecule makes it an intriguing player in host–pathogen interactions, especially in the context of changes in LPS structure or even in its loss by certain resistant pathogens. Here, we present the ADP-heptose metabolism, outline the mechanisms of its recognition and the activation of its immunity, and summarize the role of ADP-heptose in the pathogenesis of infection. Finally, we hypothesize about the routes of the entry of this sugar into cytosol and point to emerging questions that require further research.

## 1. Introduction

The critical problem of bacterial drug resistance has been recognized by the World Health Organization (WHO), who recently identified a group of bacteria for which there is an urgent need to develop antimicrobial agents [1]. The Infectious Diseases Society of America also specified *Enterococcus faecium*, *Staphylococcus aureus*, *Klebsiella pneumoniae*, *Acinetobacter baumannii*, *Pseudomonas aeruginosa*, and *Enterobacter* spp. with the acronym ESKAPE—from the first letters of the names of individual bacterial species—which is a means to communicate how these bacteria “escape” the effects of antibacterial drugs [2]. Due to the fact that the repertoire of effective antibiotics against infection with Gram-negative bacteria is being exhausted, novel therapeutic approaches including immunotherapies are emerging as important treatment options. The key for the discovery of new immuno-therapeutics is an in-depth understanding of the microbial pathogenesis and host–pathogen interactions. Here, we focus on the metabolite that appears to have immunomodulatory potential: ADP-L-glycero-β-D-manno-heptose (ADP-heptose). This metabolite has been identified as a novel pathogen-associated molecular pattern (PAMP) of Gram-negative bacteria [3,4,5,6]. ADP-heptose, which is an intermediate carbohydrate metabolite of the lipopolysaccharide (LPS) biosynthesis pathway, was not only shown to trigger the host’s immune response but also is an important factor for the fitness of Gram-negative bacteria [3,4,5,7,8,9]. Recognition of the bacterial immunomodulatory metabolites is critical to understand how the host cells detect pathogens and initiate an immune response. Moreover, this knowledge can be used for the development of antimicrobials targeting these substrates in the future. As bacteria with a “deep-rough” version of LPS are more sensitive to hydrophobic antibiotics [10] and blocking the heptose pathway also results in reduced virulence [11], there is a great need to investigate inhibitors of the LPS biosynthesis pathway (including ADP-heptose). Therefore, in this paper, we review the role of ADP-heptose in the infection process in vitro and in vivo and summarize the current understanding of the mechanism of ADP-heptose influence on various biological processes of host cells.

## 2. LPS and Mechanisms of Its Recognition by the Immune System

LPS is a glycolipid of the outer membrane of Gram-negative bacteria with a structure that is characterized by the presence of three main regions: lipid A, the core region, and O-antigen (O-polisacharide). The LPS layer is impermeable to various types of toxins, proteases, lysozyme, detergents, and hydrophobic antibacterial compounds. The LPS released in large amounts as a result of the lysis of bacterial cells can become an endotoxin, causing many diseases, including sepsis. Thus, LPS is a long-known bacterial virulence factor responsible for pathogenesis [12,13]. The main factor responsible for the immunomodulatory properties of LPS is lipid A, which is the conserved, hydrophobic part of LPS inserted into the outer membrane, consisting of a phosphorylated glucosamine dimer with fatty acids attached. The outermost domain of LPS, O-antigen, which is highly variable across bacterial strains, is responsible for resistance to the bactericidal effect of the complement system. The exposure of O-antigen, consisting of a repetitive glycan polymer on the outer surface of the bacterial cell, makes it antigenic and therefore a target for recognition by host antibodies. The core domain, in turn, is anchored between the O-antigen and lipid A; further, it is divided into the inner core and the outer core [14,15]. The inner core, highly conserved across bacterial strains, contains 3-deoxy-D-manno-oct-2-ulosonic acid (keto-deoxy-octulosonate (Kdo)) and the sugar L-*glycero*-D-*manno*-heptose (L,D-heptose), with the possibility of phosphorylation [14,16]. The outer core, in turn, consists of hexose residues, such as D-glucose, D-galactose, D-mannose, or heptose, including D,D-heptose. Certain species of bacteria, such as *K. pneumoniae* and *Helicobacter pylori*, contain L,D-heptose in both the inner and outer cores [17,18]. LPS has a complete core structure and lipid A is called “rough LPS”, whereas LPS containing only lipid A and an inner core (complete or partial) is called “deep-rough LPS”. The complete LPS molecule is considered a “smooth type LPS” [19]. In certain types of bacteria, such as *Neisseria* spp. and *Haemophilus* spp., and in certain species of bacteria, such as *A. baumannii*, the O-chain is replaced by a short oligosaccharide, and this type of LPS molecule is called LOS (lipooligosaccharide) [20,21,22,23]. Each of these forms of LPS is responsible for activating the host’s innate system, but bacteria with an incomplete structure of LPS are more sensitive to hostile environments such as those found in detergents or antibiotics [10]. Moreover, it has been shown that the ΔWaaC (heptosyltransferase) mutant of *Shigella flexneri*, which has an incomplete inner core of LPS (deep-rough mutant) due to impaired heptose synthesis, is characterized by increased biofilm formation, adhesiveness, and invasiveness to human epithelial cells [24]. Interestingly, despite the increase in the ability to adhere, no colonization was observed in the intestine of the studied infected guinea pigs, indicating that adhesiveness is not a sufficient factor to compensate for the decreased viability due to the lack of LPS fragments. Thus, the increased hydrophobicity of the cell surface associated with the lack of the LPS inner core was positively related to the adhesiveness of *S. flexneri*. The treatment strategy of targeting the oligosaccharide core is considered an alternative strategy to antimicrobial-resistant bacterial infections [24], which indicates an urgent need to study the synthesis pathway of the oligosaccharide core, including ADP-heptose. Additionally, LOS contributes to the virulence of certain bacteria by means of molecular mimicry due to antigenic diversity, resulting in the evasion of the host immune defense [20]. Nevertheless, each type—“smooth”, “rough”, and “deep-rough”—of LPS can act as a PAMP, thereby activating the innate immune responses in various cell types by the stimulation of the Toll-like receptor 4 (TLR4), resulting in the release of pro-inflammatory cytokines and type I interferons [25]. Briefly, the recognition of circulating LPS via the TLR4-mediated pathway requires the adapter protein MD-2 [26], which directly binds lipid A [27,28]. This complex binds non-covalently to TLR4, which, in turn, results in an intracellular signaling cascade, leading to the secretion of pro-inflammatory cytokines and interferons, thereby inducing an inflammatory response [29]. Intracellular signaling may follow the TLR4/MyD88 (myeloid differentiation primary response gene 88)/NF-kB (nuclear factor kappa-light-chain-enhancer of activated B cells) or TLR4/TRIF (TIR-domain-containing adapter-inducing interferon-β)/IRF3 (Interferon regulatory factor 3) pathway. The first-mentioned pathway is initiated by the LPS/MD-2/TLR4 complex located on the cell membrane, while the second-mentioned pathway begins after complex internalization into endosomes [30]. Additionally, the LPS from intracellular bacteria, or that derived from extracellular bacteria that is endocytosed or transported into the cytosol of the host cells, can be recognized by intracellular receptors as caspase-4/11 (caspase-4 in humans, caspase-11 in mice) [31,32]. Caspase-4/11 binds directly to lipid A, although the mechanism of this interaction is barely understood [31]. The activation of caspase-4/11 typically leads to the activation of the canonical NLRP3 inflammasome and cell death in the form of pyroptosis [32]. Interestingly, compared with the TLR4/MD-2 complex, these inflammatory caspases can respond to a much wider range of lipid A variants in terms of the number and length of lipid chains attached to the diglucosamine skeleton [31].

## 3. ADP-Heptose Metabolism

**Figure 1:**
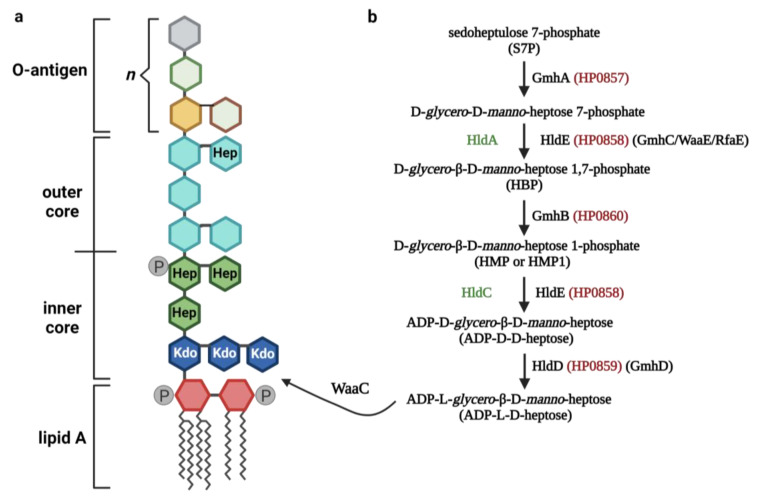
Biosynthesis of lipopolysaccharide (LPS). (**a**) Structure of LPS. (**b**) Schematic illustration of the ADP-heptose synthesis pathway: GmhA (sedoheptulose 7-phosphate isomerase); bifunctional HldE (D-β-D-heptose 7-phosphate kinase and D-β-D-heptose 1-phosphate adenylyltransferase); GmhB (D-*glycero*-α,β-D-manno-heptose 1,7-bisphosphate 7-phosphatase); HldD (ADP-D-β-D-heptose epimerase); and the enzymes present in the *Neisseria* species. In green: HldA (β-D-heptose-7-phosphate kinase) and HldC (β-D-heptose-1-phosphate adenosyltransferase). The nomenclature for genes coding enzymes present in *H. pylori* is shown in red, and the equivalent names for enzymes are shown on the far right of the pathway.

ADP-heptose (ADP-L-D-heptose) and its isomer, ADP-D-*glycero*-β-D-*manno*-heptose (ADP-D-D-heptose), are intermediate products of the inner core and outer core LPS biosynthesis, respectively [33]. ADP-heptose is generated in a five-step pathway (Figure 1) [34]. In *E. coli*, the first step of its biosynthesis involves the ketose-aldose isomerase GmhA activity, which catalyzes the isomerization of the sedoheptulose 7-phosphate (S7P) into D-*glycero*-D-*manno*-heptose 7-phosphate. This molecule is then phosphorylated by the enzyme HldE (also referred to as GmhC, WaaE, and RfaE), forming D-*glycero*-β-D-*manno*-heptose 1,7-phosphate (HBP). This, in turn, is dephosphorylated at the C-7 position by the phosphatase GmhB, assembling D-*glycero*-β-D-*manno*-heptose 1-phosphate (HMP or HMP1). The bifunctional enzyme HldE transforms HMP to form ADP-D-D-heptose by adenylation. This sugar is then converted to ADP-L-*glycero*-β-D-*manno*-heptose (ADP-L-D-heptose) via epimerization by the HldD enzyme (GmhD, WaaD, RfaD) [35,36]. In certain bacteria species, such as *Neisseria meningitidis* and *Ralstonia eutropha*, the functions of the HldE are replaced by the HldA (as the β-D-heptose-7-phosphate kinase)and HldC (β-D-heptose-1-phosphate adenosyltransferase) enzymes [36,37]. The final form of heptose that is synthesized in this pathway is integrated by the heptylotransferase WaaC into the core region of the LPS, leading to the Kdo2-lipidA formation, thus creating the basic final structure of the LPS (without O-antigen structure) [36,38]. Interestingly, there are exceptions to the presence of the ADP-heptose pathway in Gram-negative bacteria. Firstly, *Streptomyces fimbriatus* and *Streptomyces hygroscopicus*, despite being Gram-positive bacteria, synthesize ADP-heptose, which serves them as a precursor for the biosynthesis of the secondary metabolites septacidin and hygromycin B [39]. Secondly, certain bacteria, such as *Moraxella* sp., *Rhizobium* sp., *Francisiella* sp., *Legionella* sp., and *Brucella* sp., do not possess the ability to biosynthesize this sugar [34]. Colistin, an antibiotic binding to the lipid A of LPS [40], is often the last resort for the treatment of infections caused by the MDR Gram-negative pathogens [41]. The synergistic effect of colistin and doripenem in vitro has been proven against the group of ESKAPE pathogens (*P. aeruginosa*, *K. pneumoniae,* and *A. baumannii*) [42,43,44,45]. Combination therapy against *A. baumannii* reduced the levels of three metabolites of the pentose phosphate pathway (PPP), namely sedoheptulose 7-phosphate (S7P), D-ribose 5-phosphate, and D-erythrose 4-phosphate [46]. The abovementioned studies suggest that these antibiotics interfere with ADP-heptose biosynthesis in *A. baumannii* by inhibiting PPP. This includes D-sedoheptulose-7-phosphate, which is the initial metabolite involved in the internal biosynthesis of LPS and is the precursor of ADP-heptose. Furthermore, during infection, bacteria need to undergo rapid replication, which is associated with the need for a significant synthesis of LPS. Hence, bacteria devoid of the enzymes critical for the ADP-heptose pathway, such as GmhB, produce a significantly lesser amount of LPS. This, in turn, may result in increased susceptibility to killing by host defense mechanisms (including phagocytosis) [7]. Interestingly, the effects of GmhB activity on bacteria fitness are site specific and are the most significant in the spleen [7]. These facts make the ADP-heptose pathway a potentially promising drug-targeting candidate, which indicates an urgent need to investigate it further.

## 4. The Road from Recognition of the Role of HMP and HBP to the Discovery of ADP-Heptose as a Novel PAMP

The first studies suggesting the involvement of the carbohydrate of the inner core LPS biosynthesis pathway in the induction of the immune response identified HMP as the activating molecule (PAMP). This *Neisseria-gonorrhoeae*-derived HMP-activated human CD4^+^ T cell expresses the pro-inflammatory cytokines (IL-8 and TNF) via the NF-κB-dependent pathway [47]. The genome-wide mutagenesis of *N. gonorrhoeae* revealed that mutation in the hldA gene (resulting in a deep-rough LOS form) inhibited the bacterial induction of HIV-1 expression and thus viral replication in the CD4^+^ T cells. These results indicate the agonistic effect of HMP from *N. gonorrhoeae* on the immune system [47]. Subsequent results from the same group, which utilized a mouse infection model with *N. meningitidis*, demonstrated that the HBP rather than HMP—an earlier intermediate metabolite in the inner core LPS synthesis pathway—is responsible for the induction of innate and adaptive immune responses, as is evidenced by the increase in the production of local cytokines, neutrophil recruitment, and specific immunoglobulin M (IgM) and immunoglobulin G (IgG) (particularly IgG2a, b, and IgG3) production. That work demonstrated in vitro that recognition of HBP triggers a phosphorylation-dependent oligomerization of the TRAF-interacting protein with a forkhead-associated domain (TIFA) in an NF-κB-dependent manner. The key enzyme responsible for eliciting such an innate immune response also turned out to be HldA (an enzyme converting D-*glycero*-D-*manno*-heptose 7-phosphate into HBP); meanwhile, other enzymes of the inner core LPS biosynthetic pathway, such as GmhB and HldD, were not involved in the activation of NF-κB. It has also been shown that extracellular HBP also induced an inflammatory response both in vitro and in vivo, suggesting it possesses PAMP properties [48]. HBP has also been confirmed as a stimulant of the innate immune response (including inflammatory cytokines) following *Shigella flexneri*, *Salmonella typhimurium*, and *Helicobacter pylori* infections via the activation of the NF-κB/TIFA/TRAF6 axis [8,9,33,49]. Interestingly, alpha-kinase 1 (ALPK1) was thus identified as the sensor responsible for TIFA oligomerization [8,9]. Then, using the human models of embryonic kidney-derived HEK293T and colonic epithelial HCT116 cells, the activation of NF-κB by HMP in a TIFA-dependent manner, similar to HBP [50], was confirmed. However, it remained unclear whether HBP is a PAMP, especially due to the fact that its detecting pattern recognition receptor (PRR) has not yet been identified. Thus far, Zhou et al. identified ADP-heptose as an immunomodulator and a PAMP and discovered ALPK1 as the PRR responsible for sensing this carbohydrate [4]. These authors proved that ADP-heptose, but no other heptose pathway metabolites, can exclusively enter the host cytosol to activate the NF-κB/ALPK1/TIFA axis [4]. In contrast, HBP must be converted into ADP-heptose 7-P by the host cells’ adenylyltransferases (such as nicotinamide nucleotide adenylyltransferases) to gain the potential to activate the ALPK1 kinase. However, this effect is 100-fold less potent than it is for ADP-heptose (and even lesser for HMP) [4]. Immunomodulatory properties were found not only in ADP-L-D-heptose (ADP-heptose) but also in ADP-D-D-heptose) [4,51].

## 5. Mechanisms of Activation of the ALPK1/TIFA/NF-κB Axis by ADP-Heptose

**Figure 2:**
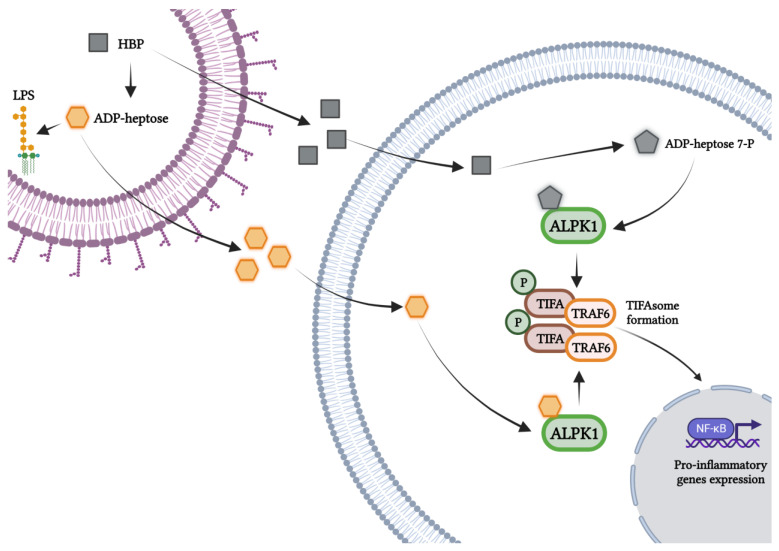
The mechanisms of activation of the host immune responses by bacteria-derived ADP-L-*glycero*-β-D-*manno*-heptose (ADP-heptose) and D-*glycero*-β-D-*manno*-heptose 1,7-phosphate (HBP). The intracellular location of HBP and ADP-heptose triggers the activation of the ALPK1/TIFA/NF-κB axis.

Accumulating data drives increases the recognition of the alpha-protein kinase 1 (ALPK1) as one of the master players in cellular processes [52,53] and also in the pathogenesis of cancer [54], recurrent periodic fever [55], ROSAH (retinal dystrophy, optic nerve edema, splenomegaly, anhidrosis, and headache) syndrome [56], and type 2 diabetes mellitus [57]. This serine/threonine kinase, a member of the alpha kinase family, has no homology in its catalytic domains to conventional kinases. This protein has a molecular weight of 139 kDa and has an N-terminal alpha-helical domain, an unstructured linker region, and a C-terminal domain [52]. Only recently, a new function of ALPK1 as a PRR in innate immunity during infection with Gram-negative bacteria was discovered (Figure 2) [4,8] and reviewed [34,58]. The catalytic activity of this protein controls the oligomerization of the tumor necrosis factor receptor associated factor-interacting protein with a forkhead-associated domain (TIFA) and the TNF receptor-associated factor 6 (TRAF6). TIFA, an adapter protein (20 kDa), was first identified as a TRAF2- and TRAF6-binding protein associated with NF-κB activation [59,60]. TIFA undergoes massive self-oligomerization (the formation of TIFAsomes) due to the phosphorylation of the threonine at position 9 by ALPK1, which is, in turn, recognized by the forkhead-associated (FHA) domain [4,8,9,61,62]. Recent research has also proved that ALPK1 phosphorylates TIFA at T177 as well as at T9 [51] and also weakly phosphorylates TIFA at T2, T12, and T19 [62]. Furthermore, ALPK1 undergoes autophosphorylation [51,62] in response to the ADP-heptose sensing that is derived from *S. flexneri* and *H. pylori* [62]. Subsequently, as in the case of the recognition of LPS via TLR4 [30], this drives the oligomerization and ubiquitination of TRAF6, which is constitutively bound to TIFA. The process of TAK1 (TGFβ-activated kinase 1), NEMO (NF-κB essential modulator), and IKK (IkappaB kinase) activation results in the activation of the NF-κB transcription factor and thus the up-regulation of the expression of the inflammatory genes (including cytokines) associated with the NF-κB signaling pathway (TNF, IL-1β, IL-6, and IL-8) [61]. More specifically, ADP-heptose is a stimulator of the formation of ubiquitin chains linked to Lys63 and Met1 to activate TAK1 and canonical IKK complexes, respectively. The redundant E3 ligases, TRAF6 and c-IAP1, form ubiquitin chains linked to Lys63, which are attached to TRAF6, TRAF2, and c-IAP1. In addition, c-IAP1 is recruited to TIFA by TRAF2. This sugar also influences the activation of the TBK1 kinase through the TAK1-independent pathway, requiring TRAF2 and TRAF6. Additionally, a mutation in Asp T177 (located within the TRAF6-binding motif) has been shown to prevent TRAF6 binding, but not TRAF2, and, as the authors indicate, this may play a role in limiting ADP-heptose signaling. Collectively, the ADP-heptose interaction in cells is co-controlled by TRAF2/c-IAP1 and TRAF6 [51].

The involvement of the TIFA protein in triggering immune response has been identified during bacterial infection with *S. flexneri*, *S. typhimurium*, *N. menengitidis* [5,8,63], *H. pylori* [6,9,33,49], *Citrobacter rodentium* [64], *Y. pseudotuberculosis* [4], and *Campylobacter jejuni* [3]. Analysis of the crystal structure of ALPK1 revealed that the ADP-heptose present in the inner core LPS of Gram-negative bacteria binds to its N-terminal kinase domain. ADP-heptose binds to a pocket on the concave side of the right-hand solenoid (composed of 18 alpha-helices, of which 14 are connected in 7 antiparallel pairs) and several residues of ALPK1 interact with ADP-heptose [4].

## 6. Biological Effects of ADP-Heptose Recognition

The discovery that ADP-heptose is a bacteria-derived metabolite that is recognized by ALPK1 and that it subsequently leads to the activation of the innate immune response is potentially a fundamental finding for a better understanding of the host–pathogen interactions. Activation of the NF-κB transcription factor by ADP-heptose through the ALPK1/TIFA pathway induces the expression of inflammatory genes during infections with pathogens such as *S. flexneri* [5,8], *S. typhimurium*, *N. meningitidis* [8], *Y. pseudotuberculosis* [4], *H. pylori* [9], and *C. jejuni* [3] (Figure 2).

In vivo studies have shown that the administration of ADP-heptose in murine models increased the concentration of inflammatory cytokines and chemokines controlled by NF-κB, such as IL-6, TNF, IP-10, MCP-1, MCP-3, IFNγ, GM-CSF, MIP-1α, MIP-1β, and RANTES, which also increased GRO-α, IP-10, and MCP-1 in serum and induced the recruitment of neutrophils [4]. Neither of these effects occurred after the injection of HBP [4]. The involvement of ALPK1 in ADP-heptose recognition was confirmed using the murine pneumonia model with *Burkholderia cenocepacia*. The involvement of this kinase in triggering inflammatory responses in the lungs was manifested by the up-regulation of MCP-3, GM-CSF, MIP-1α/-β, and RANTES in wild-type mice but not in the Alpk1-deficient (Alpk1 −/−) mice [4]. In addition, the Alpk1 deficiency resulted in a higher burden of *B. cenocepacia* in the murine lungs when compared with the lungs of wild-type mice [4]. Confirmation that ADP-heptose, but not HBP, is involved in the interaction between the heptose pathway molecules and the host response was demonstrated in vitro by the screening of a library of *Y. pseudotuberculosis* transposon mutants [4]. The ΔhldE mutant, via the transposon insertion into the hldE gene, was unable to activate NF-κB, while GmhB or hldD deletion had no effect on the activation of this nuclear factor. Interestingly, the GmhA deletion phenocopied the ΔhldE strain, which could indicate the involvement of *Y. pseudotuberculosis* HBP in the induction of NF-κB [4], which is similar to the results regarding *N. meningitidis* [48]. However, among these mutants, only the ΔGmhB supported ADP-heptose-dependent autotransporter heptosylation. In addition, as already mentioned, among the heptose metabolites, ADP-heptose is the only one able to enter the host cytosol and the only one to trigger NF-κB signaling [4]. Moreover, a comparison of the in vitro effects of chemically synthesized ADP-heptose and HBP molecules revealed higher levels of mRNA and protein of IL-8 upon stimulation with the former. Furthermore, intracellular introduction of ADP-heptose metabolites by electroporation induced more potent NF-κB activation and IL-8 production than the extracellular addition of the compounds. Meanwhile, extracellular HBP did not activate NF-κB at all [4]. This, in turn, highlights the need for HBP intracellular transfer in previous studies [4,9,48] and indicates the strong immunomodulatory properties of ADP-heptose. Additionally, in vitro studies on *Y. pseudotuberculosis* infection showed that ALPK1-dependent phosphorylation of TIFA at T9 is required for the formation of TIFAsomes and the activation of NF-κB [4].

Originally, in the studies on gastric pathogen *H. pylori*, HBP was identified as an intermediate in the synthesis of the inner core of LPS that activated the canonical NF-κB pathways [9,33,49]. The key mediators of NF-κB activation in human gastric epithelial cells turned out to be ALPK1 and TIFA [9]. However, later attempts to identify HBP in *H. pylori* lysates showed low amounts of this sugar and, unexpectedly, the ADP-heptose was identified as the novel, potent activating factor of the NF-κB/ALPK1/TIFA axis as the dominant PAMP in the lysates [6]. The discovery that ADP-heptose is indeed the ligand for ALPK1 was published concurrently with studies by Zhou et al. [4]. The *H. pylori* P12 mutants of the ADP-heptose synthesis pathway (GmhA, RfaE, and GmhB) were unable to induce NF-κB-dependent IL-8 expression, unlike the wild-type *H. pylori* P12 strain. Additionally, ADP-heptose could be detected (by liquid chromatography–mass spectrometry, LC-MS) only in bacteria without the abovementioned mutations. The ADP-heptose-induced activation of NF-κB was completely abolished in ALPK1 and TIFA knockout AGS (human gastric adenocarcinoma cell-line) cells. Interestingly, it was observed that the concentration of ADP-heptose, which is capable of activating NF-κB, is approximately 100-fold lower than the concentration of HBP. Finally, the activation of the ALPK1/TIFA axis by this heptose was confirmed by the observation of the formation of TIFAsomes; the induction of TIFAsomes was significantly weaker in HBP-transfected cells than in ADP-heptose-transfected or *H. pylori*-infected cells [6]. Later studies suggested that the early phagocytic cell activation, including NF-κB activation, and IL-8 secretion and maturation in the *H. pylori*-positive stomach are strongly dependent on ADP-heptose biosynthesis and are partly correlated with active cagPAI (i.e., the cag pathogenicity island, which encodes a membrane-spanning secretion system of the type IV, CagT4SS) [65]. Pure ADP-heptose, but also purified *H. pylori* lysates with the exclusion of other PAMPs, have the potential to induce immune response in THP-1 (human monocytic cell line) cells and human primary monocytes/macrophages. This effect was abolished in the absence of the TIFA protein [65]. In addition, since *H. pylori* is capable of activating epithelial cells through other innate immune pathways, including TLR2, TLR9, NOD1, and NLRP3, the concentration and amount of transcript of the IL-1β cytokine, a marker of proinflammatory signaling pathways, were also determined. NF-κB activation, and also IL-1β release, during the *H. pylori* infection of THP-1 cells was dependent on the presence of ADP-heptose; the heptose biosynthesis mutant, ΔhldE, resulted in a significant impairment in the induction of NF-κB activation [65]. Intriguingly, the secretion of IL-1β by THP-1 cells infected with live *H. pylori* was not associated with the activation of the NLRP3 inflammasome [65], which is a major intracellular sensor of innate immunity, and which is also activated in macrophages by this bacterium [66,67]. Additionally, heptose-pathway mutants and cagPAI mutants did not significantly differ in the level of IL-1β secretion in NLRP3-deficient THP-1 cells when compared with wild-type strains. Thus, *H. pylori*-derived ADP-heptose does not evoke NLRP3 inflammasome activation, which seems to be unaffected by the ALPK1-TIFA axis [65]. Nevertheless, TIFA, which is also a receptor of metabolites of the ADP-heptose pathway, has the ability to up-regulate the NLRP3 inflammasome in an NF-κB-dependent manner [68]. It has been also proven that GmhB (the HP0860 protein) (dephosphorylating HBP and yielding HMP1, which is further converted into ADP-heptose) is the key enzyme of *H. pylori* that is involved in the inner core LPS biosynthesis pathway and is responsible for bacterial virulence [69]. The HP0860 mutant showed less ability to adhere to epithelial cells, increased sensitivity to hydrophobic novobiocin, and, ultimately, less virulence [69]. Interestingly, in comparison with the HP0857 (GmhA) knockout (KO) mutant, GmhB KO was characterized by a retained ability to induce the secretion of IL-8, suggesting the activity of another enzyme responsible for compensating the loss of GmhB activity [69] or the ability of HBP to activate ALPK1/TIFA/NF-κB signaling. Moreover, the disruption of the HP0860 gene affected the sorting of proteins (which almost resulted in the absence of the virulence factor—CagA) into the outer membrane vehicles (OMVs) [69]. This finding is consistent with previous studies indicating the role of the LPS structure in directing the protein-sorting mechanism during OMV formation [70]. The necessity of the enzymes GmhA (HP0857 protein) and HldD (HP0859 protein) for *H. pylori* survival and high virulence was also identified by the same authors [71,72].

The much lower endotoxicity of *H. pylori* when compared with *E. coli* [73] is related to the structure of its LPS and is characterized by three variations within lipid A, which translates into the ability for immune escape and the ability to reduce the binding of antimicrobial peptides (including polymyxin B) and to avoid detection by TLR [74]. Therefore, studies on the ADP-heptose pathway in the pathogenesis of *H. pylori* are of great interest, especially since the role of TLRs (TLR4/TLR2) in the detection and activation of the immune response by *H. pylori* is controversial [74].

Studies on the factors for the lung persistence of *K. pneumoniae* are required for the development of subsequent secondary bacteremia. These studies have revealed that the ADP-heptose synthesis pathway related GmhB is crucial for the survival of bacteria in the blood but not in the lungs [7]. More specifically, data from multiple infection models identified GmhB as an important factor for lung dissemination and bloodstream survival. Moreover, the GmhB mutant in the direct bacteremia model displayed a significant fitness defect in the spleen and liver, unlike in the lungs. Interestingly, in the model of bacteremic pneumonia induced by *K. pneumoniae*, GmhB did not modulate lung inflammatory responses [7]. Monocytes and neutrophils were recruited to the lungs during *K. pneumoniae* infection, but the number of alveolar macrophages, eosinophils, and dendritic cells did not change. Additionally, a reduced number of monocyte-myeloid-derived suppressor cells (M-MDSC) after infection of *K. pneumoniae* (both wt/KPPR1 and GmhB mutant) was observed, regardless of the presence of GmhB [7]. The levels of inflammatory cytokines associated with the ALPK1/TIFA/NF-κB axis [4] increased after infection with *K. pneumoniae,* but this was also independent of GmhB. As the GmhB mutant did not show reduced survival in human nor mouse serum when compared with the wild-type strain, ex vivo competition assays in uninfected murine spleen and liver homogenates were performed. GmhB was essential for full fitness in the spleen homogenate but was not required for liver and lung fitness [7]. These data indicate that the presence of the GmhB enzyme increases survival in the bloodstream by mediating spleen fitness. In the murine bacteremia models, GmhB was also required for *E. coli* and *Citrobacter freundii* to survive in the spleen and liver, which highlights the highly conserved function of GdhB among *Enterobacterales* [7].

Garcia-Weber et al. [62] proved that ALPK1 undergoes autophosphorylation in response to infection by these pathogens and that the thiophosphorylation status of TIFA and ALPK1 increased along with increasing pathogen load, which persisted for several hours post-infection. Given that these bacteria are intracellular pathogens, this effect may be due to sustained ADP-heptose release through extensive bacterial replication, residual bacterial lysis, or ADP-heptose secretion by the T3SS secretion system [62,63]. The mutation of the hldE gene in *H. pylori* abolished the phosphorylation of ALPK1 and TIFA observed in wild strains of both *S. flexneri* and *H. pylori* [62], thus confirming the previous study [6] and the results obtained for *Y. pseudotuberculosis* [4].

Impairment of the heptose biosynthesis pathway by a deletion of the hldE gene in *C. jejuni* also highlighted the role of ADP-heptose (the strongest activator, but HBP and ADP-heptose-7P may also be involved) as the causative agent of ALPK1/NF-κB axis activation in HeLa 57A cells and intestinal cells [3]. This Gram-negative bacterium causes bloody diarrhea by inducing a strong pro-inflammatory response and by releasing a wide range of cytokines, chemokines, and effector molecules. ADP-heptose appears to be a potent PAMP, activating a wide set of pro-inflammatory genes, including CXCL8, CXCL2, TNFAIP2, TNFAIP3, CXCL2, CCL2, IL-6, and PTGS2, in Toll-like receptor (TLR1, TLR2, TLR3, TLR4, TLR5, TLR6, TLR7, and TLR9) and Nod-like receptor (NOD1 and NOD2) independent manners. A *C. jejuni*-dependent stimulation of HeLa cells strongly induced the secretion of NF-κB-dependent IL-8, which was mediated by ALPK1 (PRR) and was activated by released heptose metabolites. Additionally, an increase in the expression of genes responsible for the initiation and modulation of the inflammatory response (IRAK2, BIRC3, RELB, and NFKBIA), as well as the genes involved in other inflammation-related pathways (PTGS2, CD83, and ITGB8), both regulated by NF-κB, was observed. On the other hand, the absence of GmhB, which is the third enzyme of the ADP-heptose biosynthetic pathway (preceding the participation of HldE (HldC) as ADP transferase and thus leading to the formation of ADP-heptose), did not result in an altered effect on the regulation of NF-κB. In fact, the strain still produced a full-length LOS [3]. Moreover, the deletion of the waaF and waaC genes, which catalyze the heptose transfer to the LOS downstream of ADP-heptose synthesis, also did not reduce NF-κB activation [3]. When using the in vitro enzymatic model with sedoheptulose-7-phosphate as a substrate, and when only the combination of all three recombinant enzymes derived from *C. jejuni*—GmhA, HldE, and GmhB—were used, a strong activation of NF-κB occurred, approximately 1000-fold greater than the combination of only GmhA and HldE. That experiment elegantly demonstrated that ADP-heptose, but not other heptose, could be the dominant virulence factor among the carbohydrates [3].

HBP was firstly described as the PAMP of *S. flexneri* [8]; however, it was later discovered that chemically synthesized HBP (unlike bacterial lysate) does not initiate rapid TIFA oligomerization in epithelial cells, but only late (2 h post-treatment) oligomerization. Then, it was concluded that HBP must be processed intracellularly to induce inflammation and that the proper dominant and novel *S. flexneri* PAMP is ADP-heptose [5]. By leveraging the gene deletion analysis of the LPS biosynthetic pathway, it was later confirmed that these food poisoning bacteria trigger cytokine production (IL-4, IL-6, IL-8) in an APLK1-dependent manner [5].

Collectively, these results allow us to better understand the molecular processes that control inflammation in response to Gram-negative bacteria infections, and thus allow us to create the perspective for the development of new immunomodulatory therapies. Therefore, further studies on the mechanisms and role of ADP-heptose recognition by the ALPK1/TIFA pathway during infections with other pathogens are pending.

## 7. Intracellular Entry Routes of Bacterial Heptose

**Figure 3:**
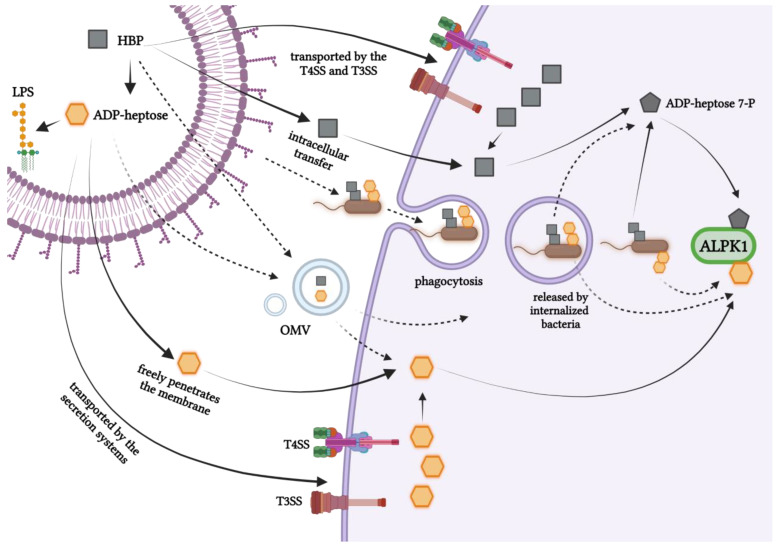
Different potential routes of ADP-heptose and HBP; the newly discovered pathogen-associated molecular patterns (PAMPs) delivery into the host cell are shown. Solid lines in arrows indicate confirmed processes, while dotted lines indicate speculative pathways.

The exact delivery mechanisms of sugar metabolites of the LPS biosynthetic pathway to host cells during bacterial infection remain unresolved (Figure 3). Does it vary depending on the species of bacteria? A positive answer to this question appears to be developing upon analysis of the results from studies on various pathogens. The mechanism by which the sugar metabolite, ADP-heptose, passes through the host cell membrane is unknown; however, it was shown that the entry of ADP-heptose does not require transfection or permeabilization engineering, unlike HBP [4]. *Y. pseudotuberculosis* was found to use the type 3 secretion system (T3SS) for this purpose [4,75]. Similarly, the up-regulation of the NF-κB-dependent cytokine expression is mediated by T3SS in bacteria such as *S. typhimurium*, enteropathogenic *E. coli*, and *Burkholderia pseudomallei* [76,77,78]. Could these bacteria also utilize T3SS to deliver ADP-heptose? T3SS typically performs the translocation and secretion of proteins and various bacterial metabolites. It is also possible that T3SS secretes ADP-heptose to a lesser extent in the extracellular medium, even before the cells are internalized [34]. *S. flexneri*, despite being an intracellular pathogen, uses T3SS to inject bacterial effector proteins and various metabolites in the early infection process, specifically when the bacteria are still outside the host cells. Thus, it is highly likely that this bacterium transfers ADP-heptose to target cells utilizing T3SS. The ALPK1/TIFA/NF-κB axis was also strongly activated during the infection of intestinal cells with *C. jejuni*, which do not possess such infective systems as T3SS or type IV secretion systems (T4SS) do. This pathogen releases ADP-heptose into the environment, and this metabolite then enters the host cells. Interestingly, it has been proven that ADP-heptose is also shed during bacterial culture, both during the logarithmic growth phase and during the stationary phase [3]. Unlike in *C. jejuni*, T4SS mediates the direct injection of ADP-heptose into the cytosol of host cells in *H. pylori* [6]. Additionally, other groups have demonstrated that NF-κB signaling in response to *H. pylori* HBP is dependent on T4SS [9,33,49]. Interestingly, although the cag bacterial island of pathogenicity, which forms the type IV secretion system (CagT4SS), was required for the *H. pylori* infection of epithelial THP-1 cells and for pure ADP-heptose alone, it was also able to strongly activate these cells and the human primary monocytes/macrophages [65]. However, as it is an intracellular pathogen, it is conceivable that the bacteria residing in internalizing vacuoles or in the cytoplasm of host cells, inject or release ADP-heptose directly into the cytoplasm [34]. The possibility of the existence of both of these non-exclusive mechanisms of ADP-heptose delivery to cells is indicated by the fact that TIFAsomes appear very quickly, within a few minutes of infection [8]. TIFAsomes are observed for several hours, suggesting that perhaps ADP-heptose, like HBP [63], is released during bacterial growth (a constant release of ADP-heptose over time was also seen in *C. jejuni* [3] or the after lysis of cytosolic bacteria). Garcia-Weber et al. considered the formation of TIFAsomes in uninfected cells, which were in contact with infected cells [8], and speculated whether ADP-heptose passes through the gap junctions, thereby activating ALPK1 in uninfected, bystander cells [34]. However, questions about the mechanisms of heptose secretion by other pathogens, which were shown to activate the immune system via the ADP-heptose, such as *K. pneumoniae*, remain to be answered. Do those pathogens use different types of secretion systems, such as T6SS? The exact mechanism by which extracellular ADP-heptose enters host cells also remains to be elucidated. One possibility may be via the dynamin-dependent endocytosis, as proposed for HBP in Jurkat cells [48]. In addition to injectisome, other virulence factors such as outer membrane vehicles (OMV) and outer membrane protein A (OmpA) [79] could also be involved in the heptose metabolite transfer. Such mechanisms could explain the redundancy of the presence of TSS among certain bacteria. Since intracellular bacteria can carry ADP-heptose while present in the internalizing vacuoles, it may be reasonable to test the role of phagocytosis in the activity of the ALPK1/TIFA/NF-κB pathway. In support of this hypothesis, it was shown that HBP from *Neisseria* sp. is released into the cytosol of epithelial cells during phagocytosis [48].

The study by Snelling et al. indicated that both ADP-D-D-heptose as well as the ADP-L-D-heptose added to the medium activated the pathway almost maximally at a concentration of 10 μM. Activation of the pathway (via activation of MAP kinases and the canonical IKK complex) in HEK293 cells or primary mouse bone marrow-derived macrophages (BMDM) could be detected in as little as 10 min and was maximal within 15–20 min after adding ADP-D-D-heptose [51]. In turn, the ADP-heptose present in the medium activated NF-κB in the THP-1 reporter cells to a high extent only after 4 h of incubation [65]. These studies suggest differential kinetics of free ADP-heptose entry into various cell types and moreover, kinetics of the pathway activation upon real infection remains to be investigated.

## 8. Puzzling Aspects of ADP-Heptose and ALPK1 in Health and Disease

In addition to its role in the recognition of Gram-negative pathogens, ALPK1 promotes intestinal homeostasis. This kinase acts as an intestinal inflammation regulator of *Helicobacter-hepaticus*-caused colitis and the associated cancer susceptibility locus in a mice model, which occurred through affecting the secretion of IL-12 and IL-23 and activation of Th1/interferon-γ [80]. Thus, the question arises of whether *H. hepaticus*, as a pathobiont, affects the regulation of ALPK1 through heptose. Then, do the host cells distinguish between pathogen-derived ADP-heptose and commensal bacteria-derived ADP-heptose?

Furthermore, a relationship between ADP-heptose recognition and cancer development has been revealed. Additionally, the DNA damage of gastric epithelial cells that was dependent on the *H. pylori* bifunctional RfaE enzyme and Cag pathogenicity island occurred only in cells that were in the S-phase of a cell cycle [81]. The DNA damage and replication stress evoked by *H. pylori* were dependent on the presence of RNA/DNA co-transcriptional hybrids (R-loops) formed in *H. pylori*-infected cells during the S-phase as a result of the cell recognition of ADP-heptose via the ALPK1/TIFA/NF-κB signaling pathway [81]. The presence of *Fusobacterium nucleatum* in the intestinal microflora of patients with colorectal cancer (CRC) promoted the formation of metastases by inducing ICAM1 expression; it also promoted the adhesion of CRC cells to the endothelium [82]. Interestingly, this was accomplished through the triggering of the ALPK1/NF-κB axis by this bacterium. In addition, overexpression of ALPK1 or ICAM1 correlated with shorter survival in CRC patients [82]. Similarly, an increased expression of ALPK1 was found to facilitate the progression of oral squamous cell carcinoma (ALPK1 has a function as an oncogene) [83]. It is therefore puzzling how, if at all, ADP-heptose and other sugar metabolites of the LPS biosynthesis pathway of the *F. nucleatum* induce the ALPK1/TIFA/NF-κB signal in the host cells.

Research on another commensal pathogen, *Akkermansia muciniphila*, revealed the involvement of the ALPK1/TIFA/TRAF6/NF-κB axis in response to the commensal metabolite with the biological properties of ADP-heptose. That metabolite, which was released by this intestinal symbiont, induced the expression of the MUC2, BIRC3, and TNFAIP3 genes that are involved in the maintenance of intestinal barrier function and Cxcl8, which encodes the pro-inflammatory cytokine IL-8 [84]. This raises a question about the effects of the commensal-derived ADP-heptose induction of the ALPK1 signaling pathway on the further response to pathogen-derived ADP-heptose and other PAMPs. Altogether, we speculate that the ADP-heptose-triggered activation of the immune system by the commensals and pathogens plays an important role in the maintenance of mucosal immunity.

The associations of mutations in the ALPK1 gene with inflammatory syndromes and cancer, especially the ROSAH syndrome (the T237M and the Tyr254Cys mutant) and spiradenoma/spiradenoma (the V1092A mutant), were revealed recently [54,85]. The ALPK1 mutations related to these disease entities exhibited increased spontaneous kinase activity and constitutive TIFAsome assembly, which could be further boosted by ADP-heptose [62]. Cohort studies showed that untreated patients with the Thr237Met and the Tyr254Cys mutations (ROSAH syndrome) had recurrent elevations of not only CRP (C reactive protein) but also of pro-inflammatory cytokines and chemokines, including TNF, IL-6, CCL2 (MCP-1), soluble IL-2 alpha receptor, and IL-10, CXCL10 (IP-10) in plasma and CXCL1 in serum (GRO-alpha). However, there was no change in the amount of intracellular IFN-gamma, TNF, or IL-4 after the stimulation of peripheral blood cells. Anti-TNF and anti-IL-1 anti-cytokine therapies inhibited systemic inflammation and improved quality of life, but anti-IL-6 (tocilizumab) was found to be the only therapy to reduce intraocular inflammation. ADP-heptose stimulation of fibroblasts derived from patients with the T237M mutation but not from healthy controls led to increased phosphorylation of kinases that were characteristic of the active NF-κB canonical pathway (IκBα, IKKα/β, MAP p38, and JNK) [85]. Moreover, the T237Mand the Tyr254Cys mutations were found to be associated with increased signal transducers and activators of transcription (STAT1) phosphorylation and the expression of interferon-regulated genes [85]. Interestingly, p.V1092A spiradenoma mutants have also been shown to have the constitutive activity of ALPK1 and NF-κB signaling in the absence of ADP-heptose [54].

ALPK1 has also been noted as a gene related to the susceptibility to type 2 diabetes mellitus [57]. However, the rs2074379 (M732I) and rs2074388 (G565D) ALPK1 variants were significantly associated not only with the prevalence of type 2 diabetes [57] but also with chronic kidney disease [86] and gout [87]. The ALPK1 missense variant rs2074388 (G565D) in exon 11 was also found in colon cancer patients [88]. These results indicate that this kinase may be involved in many biological processes beyond its role in immunity to Gram-negative bacteria. In addition to ALPK1 being involved in the progression of breast, lung, colorectal, oral, and skin cancers, as well as lymphoblastic leukemia, ALPK1 mutations are associated with gout, diabetes, and chronic kidney disease, which are thought to be related to breast, lung, colon, urinary tract, pancreas, and endometrium cancers, as well as lymphoblastic leukemia [89]. Altogether, these results showed an ADP-heptose-dependent mechanism promoting the malignant transformation of gastric cells. Moreover, in *H. pylori*-infected gastric cells, TIFA performed a dual function: it was involved in both the classic and alternative NF-κB signaling (via the TRAF6 and TRAF2 axes, respectively) [90].

## 9. Conclusions

The sensing of ADP-heptose, a metabolite of the inner core LPS biosynthesis in Gram-negative bacteria (identified as PAMP), by ALPK1 (PRR), further leading to the activation of TIFA and NF-κB, represents a newly discovered but still unknown mechanism. A thorough knowledge and understanding of this pathway in the context of bacterial infections could help with understanding pathogenic invasion and could indicate a new approach to the development of antimicrobial strategies through immunotherapy, including the development of novel vaccine adjuvants. Since ADP-heptose, as a small molecular weight molecule, is a very conservative PAMP, its role in immunity function and its therapeutic modulation is tempting to utilize. Its importance can be increased in the context of changes in the LPS structure and even its loss by certain MDR pathogens (i.e., *A. baumannii* [91,92]). However, the current knowledge is partial and generates multiple unanswered questions. For instance, we do not know whether and how the host distinguishes between the commensal flora-derived and pathogen-derived ADP-heptose. Additionally, there is a lack of general understanding of the routes of ADP-heptose delivery to the cytosol. In turn, a question arises of whether the host can distinguish the signal coming from live and replicating bacteria and colonization. Undoubtedly, there are currently gaps in the knowledge of the effects of ESKAPE-pathogen-derived ADP-heptose on the host immune response that require further investigation.

## Data Availability

The raw data supporting the conclusions of this manuscript will be made available by the authors, without undue reservation, to any qualified researcher.

## Abbreviations

ADP-heptose	ADP-L-*glycero*-β-D-*manno*-heptose
HBP	D-glycero-β-D-manno-heptose 1,7-phosphate
HMP	D-glycero-β-D-manno-heptose 1-phosphate
Kdo	3-Deoxy-D-manno-oct-2-ulosonic acid
LPS	Lipopolysaccharide
LOS	Lipooligosacharide
TLR4	Toll-like receptor 4
PAMP	Pathogen-associated molecular pattern
PRR	Pattern recognition receptor
TIFA	TRAF-interacting protein with a forkhead-associated domain
ALPK1	Alpha kinase-1
MyD88	Myeloid differentiation primary response gene 88
NF-κB	Nuclear factor kappa-light-chain-enhancer of activated B cells
TRIF	TIR-domain-containing adapter-inducing interferon-β
IRF3	Interferon regulatory factor 3
